# Prognostic Impact of *BRAF* and *KRAS* Mutation in Patients with Colorectal and Appendiceal Peritoneal Metastases Scheduled for CRS and HIPEC

**DOI:** 10.1245/s10434-019-07452-2

**Published:** 2019-09-30

**Authors:** Wilhelm Graf, Peter H. Cashin, Lana Ghanipour, Malin Enblad, Johan Botling, Alexei Terman, Helgi Birgisson

**Affiliations:** 1 Department of Surgical Sciences, Akademiska sjukhuset, Uppsala University, Uppsala, Sweden; 2 Department of Immunology, Genetics and Pathology, Clinical and Experimental Pathology, Akademiska sjukhuset, Uppsala University, Uppsala, Sweden

## Abstract

**Background:**

*KRAS* and *BRAF* mutations are prognostic and predictive tools in metastatic colorectal cancer, but little is known about their prognostic value in patients scheduled for cytoreductive surgery (CRS) and hyperthermic intraperitoneal chemotherapy (HIPEC). Therefore, we analyzed the prognostic impact of *KRAS* and *BRAF* mutations in patients with peritoneal metastases scheduled for CRS and HIPEC.

**Patients and Methods:**

In a consecutive series of 399 patients scheduled for CRS and HIPEC between 2009 and 2017, 111 subjects with peritoneal metastases from primaries of the appendix, colon, or rectum were analyzed for *KRAS* mutation and 92 for *BRAF* mutation.

**Results:**

Mutation in *KRAS* was present in 51/111 (46%), and mutated *BRAF* was found in 10/92 (11%). There was no difference in overall survival between *KRAS* mutation tumors and *KRAS* wild type, whereas *BRAF* mutation was associated with short survival. No subject with *BRAF* mutation survived 2 years. On multivariate analysis, completeness of cytoreduction score (CCS, *p* = 0.000001), presence of signet cell differentiation (*p* = 0.000001), and *BRAF* mutation (*p* = 0.0021) were linked with poor prognosis.

**Conclusions:**

*BRAF* mutation is a marker of poor prognosis in patients with appendiceal and colorectal peritoneal metastases scheduled for CRS and HIPEC, whereas survival outcome in subjects with mutated *KRAS* does not differ from wild-type *KRAS*. This finding suggests that those with *BRAF* mutation should be considered for alternative treatment options.

Appropriate patient selection for cytoreductive surgery (CRS) and hyperthermic intraperitoneal chemotherapy (HIPEC) is essential for optimized results.^1^,^2^ Patient-related factors such as performance status and comorbidity must be weighed against tumor extension and localization. The importance of primary tumor origin and tumor burden as measured with the peritoneal cancer index (PCI) has been clearly demonstrated in previous studies.^3^^–^5 Molecular markers such as *KRAS* and *BRAF* mutations have been used as predictive tools for optimizing antibody treatment with epidermal growth factor receptor (EGFR) blockers, and targeted therapies against *BRAF* mutated tumors have been used in advanced melanoma and lung cancer treatments. The prognostic importance of *BRAF* mutation has varied in previous studies. One study revealed a lower risk of tumor dissemination in patients with primary colorectal cancer,^6^ but on the other hand, in the metastatic state, *BRAF* mutated tumors were linked with poor prognosis,^7^ especially if associated with microsatellite stable tumors.^8^ Molecular analyses have been applied in methodological studies,^9^,^10^ but only one study has analyzed the prognostic value of *KRAS* and *BRAF* mutations in patients with colorectal peritoneal metastases.^11^ In that study, both mutations were associated with poor prognosis.

The aim of this study is to analyze the prognostic influence of *KRAS* and *BRAF* mutations in patients with peritoneal metastasis from appendiceal or colorectal adenocarcinoma scheduled for CRS and HIPEC.

## Patients and Methods

### Patients

A total of 399 patients were scheduled for CRS and HIPEC between January 2009 and September 2017 at the Department of Surgery, University Hospital, Uppsala, Sweden. Seven patients underwent reoperations, and 58 patients were diagnosed with nonappendiceal or noncolorectal tumors and were excluded from further analysis. All remaining 334 subjects had suspected isolated peritoneal metastases and were judged as potentially curable; i.e., there were no signs of distant tumor spread at the preoperative work-up except for limited and resectable hepatic involvement. The routine work-up consisted of abdominal and thoracic computed tomography (CT) scans, colonoscopy, and routine blood tests including tumor markers, whereas laparoscopy was used in cases where extensive small bowel involvement or other signs of irresectable disease were suspected on preoperative CT scans.

There were no histologically detectable neoplastic cells in the specimens of 39 patients although pre- and intraoperative assessment suggested peritoneal metastases. These patients were also excluded, leaving a total of 295 patients relevant for analysis (Table 1). The primary tumor was colorectal cancer in 178 individuals and appendiceal neoplasms in the remaining 117. *KRAS* and *BRAF* mutation status was assessed by pyrosequencing and was performed selectively in 111/295 (38%) of the patients based on clinical indications. A total of 232 patients (79%) received HIPEC, whereas 47 cases were open and close procedures, i.e., judged inoperable, usually because of extensive small bowel involvement. Our policy is to abandon the procedure and refrain from HIPEC if there are definite signs that a completeness of cytoreduction score of 0–1 cannot be achieved.

**Table 1:** Clinical characteristics of 295 patients with colorectal and appendiceal peritoneal metastasis scheduled for CRS and HIPEC in relation to whether mutation analysis for KRAS and BRAF was performed (*n* = 111) or not (*n* = 184)

	Analyzed	Not analyzed	*p*
	(*n* = 111)	(*n* = 184)	
Age	61.5 ± 12.1	58.1 ± 12.3	0.022
Male:female	55:56	85:99	0.576
PCI	17.47 ± 10.64	18.47 ± 11.96	0.475
CC score			
0	82	115	
1	5	37	
≥ 2	24	32	0.369*
Colon cancer	84 (76)	69 (38)	0.919^+^
Right sided	50	39	
Left sided	33	26	
Unspecified	1	4	
Rectal cancer	14 (13)	11 (6)	
Appendiceal tumor	13 (12)	104 (56)	< 0.001^#^
Mucinous tumor	44 (40)	40 (22)	0.015
Signet cell cancer	24 (22)	23 (13)	0.056
CRS + HIPEC	81 (73)	151 (82)	0.089
Hepatic resection	17 (15)	13 (7)	0.038
Open–close procedure	22 (20)	25 (14)	0.210
*KRAS* mutation	51 (46)	–	
*KRAS* wild type	59 (54)	–	
*BRAF* mutation	10 (11)	–	
No *BRAF* mutation	82 (89)	–	

Data presented as mean ± SD or number (percentage)

*CCS 0–1 versus CCS > 1

^+^Colon versus rectal primary

^#^Appendiceal versus nonappendiceal primary

Sixteen subjects were not treated with HIPEC because of intraoperative complications or doubtful indication. Hepatic Glisson capsulectomy was performed in 24 patients, and formal hepatic resection was performed in 30 cases.

### Cytoreductive Surgery and HIPEC

Initially, the abdominal tumor extension was quantified using the PCI, and the ability to perform an R0 resection was assessed by examining the small bowel and other potential failure sites. The technique of cytoreduction consisted of several peritonectomies combined with omentectomy and removal of disease-affected organs, as previously described.^12^ Briefly, diathermy was used for stripping of the peritoneal layers from the abdominal wall, pelvic walls, and diaphragm. A macroscopically healthy peritoneum was left in situ (i.e., resections were performed depending on the extent of the macroscopic tumor). After CRS, the remaining amount of tumor was graded using the completeness of cytoreduction score.^13^,^14^ Immediately after cytoreduction, HIPEC was performed according to the Coliseum method. Briefly, one inflow catheter was placed centrally in the abdomen, and four closed suction drains were inserted through the lateral abdominal wall, allowing for outflow of the chemotherapy solution. Three HIPEC regimens were used: The standard regimen for colorectal primaries was oxaliplatin 460 mg/m^2^ administered over 30 min, preceded by 5-flurouracil 400 mg/m^2^ combined with calcium folinate 30 mg/m^2^ as an IV infusion. As an alternative, e.g., in case of side effects or tumor progress after previous systemic oxaliplatin treatment, irinotecan 460 mg/m^2^ was used as intraperitoneal treatment. Appendiceal tumors with mucinous peritoneal implants were treated with mitomycin 30 mg/m^2^ over 90 min as intraperitoneal treatment.

### Histopathology and Mutation Analysis

Solid tumor specimens were collected from all resection sites and fixed in 4% buffered formaldehyde. Paraffin-embedded blocks of tissue were sectioned with a microtome in 3–4-µm sections and stained with hematoxylin–eosin for routine examination. DNA was extracted from paraffin-embedded blocks, and samples with maximum tumor content were obtained by manual microdissection. The PyroMark Q24 BRAF and KRAS version 2.0 assays (Qiagen) were used to detect mutations in *BRAF* (codon 600) and *KRAS* (codons 12, 13, and 61 in exons 2 and 3) according to the manufacturer’s recommendations. *KRAS* and *BRAF* mutation status was assessed by pyrosequencing (2007–2014) or targeted next-generation sequencing (2015–2016).^15^,^16^ Results from mutation anlyses were retrieved from the pathology reports reflecting the clinical routine during the study period.

A sequence library was constructed using a Haloplex™ DiagnPanel_Colon_20160222, and sequencing was performed using a MiSeq instrument (Illumina, San Diego, CA). The analysis was performed on material from the primary tumor in 75 cases and on peritoneal metastases in 36 cases.

### Statistical Methods

Figures are presented as mean (standard deviation, SD), and differences were assessed using Student’s *t* test. Differences in proportions were assessed by Chi square test or Fisher’s exact test, where appropriate. Overall survival was calculated from date of surgery to date of death from any cause or last follow-up. Survival state was recorded using the Swedish National Population Register as of the end of 2017. Survival curves were constructed according to Kaplan–Meier and differences evaluated by log-rank test. Multivariate analysis was performed using a Cox proportional hazard procedure, and risk estimates are presented as relative risk (RR) with 95% confidence limits of RR. *p* Value < 0.05 was considered statistically significant. Statistical analyses were performed using Statistica 13 software (Palo Alto, CA). The study was approved by the ethics committee of Uppsala County.

## Results

### Clinical Variables and Histopathology

Age (*p* = 0.97) and gender (*p* = 0.39) did not affect survival. The most favorable survival was observed for patients with peritoneal metastases from an appendiceal tumor (projected 5-year survival 73%, median survival not reached); intermediate prognosis was observed for peritoneal spread of colon cancer origin (projected 5-year survival 38%, median survival 35 months), and the worst prognosis for rectal cancer origin (projected 5-year survival 22%, median survival 18 moths, *p* < 0.0001, log-rank test). Signet ring cell differentiation was associated with reduced survival (*p* = 0.0015), whereas mucinous histology was not (*p* = 0.57). Those with PCI below 20 experienced longer survival compared with the group with higher PCI (*p* = 0.0008). Survival outcome in the 25 patients who underwent liver resection as part of CRS did not differ from those without liver resection (*p* = 0.34).

### Molecular Analysis

*KRAS* mutated tumors were evenly located among the appendix, colon, and rectum, whereas *BRAF* mutated tumors were predominantly situated in the right colon (Table 2). The proportion undergoing mutation analysis was much smaller among those with an appendiceal primary tumor compared with colorectal tumors (13/117, 11% versus 98/178, 55%, *p* < 0.0001, Table 1). The 13 patients with a primary in the appendix all had invasive adenocarcinoma, mostly with poor differentiation. Three had mucinous fluid but also solid tumor, whereas 10 had solid peritoneal tumor nodules (Table 3).

**Table 2:** Characteristics of patients with peritoneal metastasis of colorectal and appendiceal origin in relation to results of mutation analysis for KRAS and BRAF

	KRAS			BRAF		
	Mutated	Wild type	*p*	Mutated	Not mutated	*p*
	(*n* = 51)	(*n *= 59)		(*n* = 10)	(*n* = 82)	
Age	61.4 ± 11.3	60.1 ± 13.1	0.555	67.9 ± 7.53	60.4 ± 12.84	0.085
Male:female	26:25	28:31		5:5	36:46	
Primary tumor						
Appendiceal	7	6	0.780*	0	10	0.594*
Right colon	22	28		7	30	
Left colon	16	16		2	29	
Colon unspec	0	1		0	1	
Rectum	6	8	0.995^+^	1	12	1.0^+^
Lymph nodes						
Not examined	24 ± 12.8	21 ± 12.0	0.191	19 ± 9.9	22 ± 12.0	0.567
Not involved	4 ± 5.5	6 ± 8.1	0.165	8 ± 11.8	5 ± 5.5	0.126
PCI	18.6 ± 11.3	17.1 ± 10.5	0.487	15.2 ± 9.2	17.3 ± 11.2	0.570
CC score						
0	41	41		7	64	
1	3	2		0	3	
≥ 2	7	16	0.137^#^	3	15	0.404^#^
Liver resection	9	8	0.744	1	12	0.003
Mucinous	23	20	0.315	3	36	0.509
Signet cell diff.	10	13	0.939	1	19	0.685

Data presented as mean ± SD or number

*Appendiceal versus nonappendiceal primary

^+^Rectal versus colon primary

^#^CCS 0–1 versus CCS > 1

**Table 3:** Histopathological characteristics of primary tumor and macroscopic appearance of peritoneal carcinomatosis in 13 patients with appendiceal tumors that were analyzed for KRAS and BRAF mutations

T-stage	N-stage	Mucinous	Differentiation	Macroscopic appearance	PCI/CCS
T3	N0 (0/18)	Mucinous	NS*	Solid implants	19/0
T4	N1(1/21)	Nonmucinous	Poor	Solid implants	7/0
T3	N1(3/15)	Nonmucinous	Signet	Solid implants	28/0
T4	N0(0/8)	Mucinous	Moderate	Solid implants	8/0
T4	N2(14/15)	Nonmucinous	Poor	Solid implants	26/0
T4	NS	Mucinous	Signet	PMP^+^-like	38/1
T4	NS	Mucinous	Moderate	PMP-like	32/0
T4	N2(10/26)	Mucinous	Signet	Solid implants	11/0
NS	NS	Mucinouss	Poor	Solid implants	34/3
T2	N0(0/19)	Mucinous	NS	PMP-like	36/0
T3	N0(0/16)	Mucinous	Moderate	Solid implants	20/3
T4	N0 (0/16)	Mucinous	Signet	Solid implants	7/0
T4	N2(12/24)	Mucinous	Signet	Solid implants	4/0

*Not stated

^+^Pseudomyxoma peritonei

**Fig. 1:**
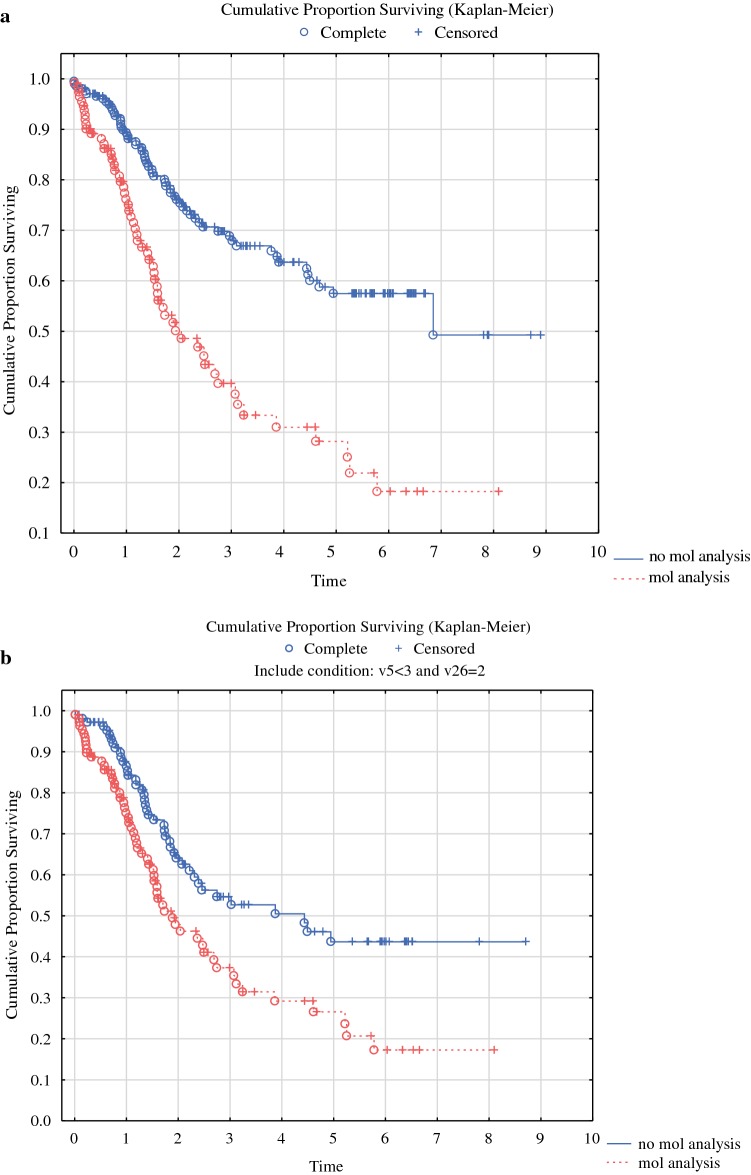
**a** Overall survival in 295 patients with peritoneal metastasis of appendiceal or colorectal origin depending on whether mutation analysis was performed or not. **b** Overall survival in 178 patients with peritoneal metastasis of colorectal origin depending on whether mutation analysis was performed or not

**Fig. 2:**
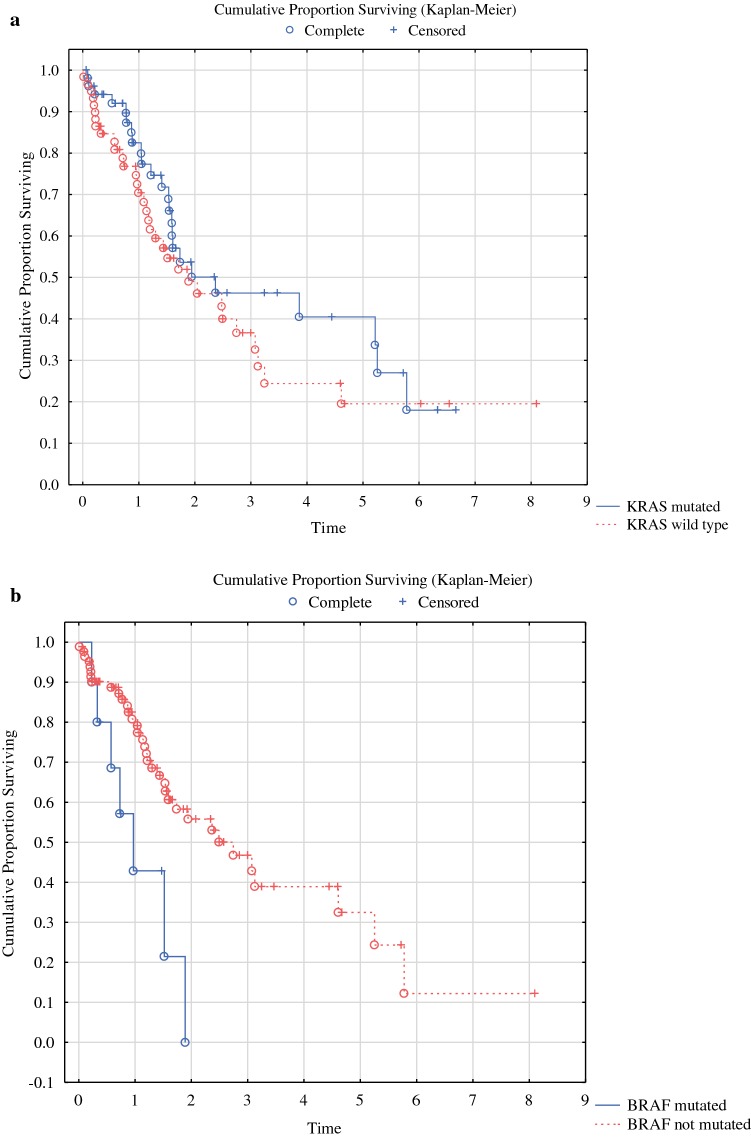
**a** Overall survival in relation to results of *KRAS* mutation analysis in 111 patients with peritoneal metastasis of colorectal or appendiceal origin. **b** Overall survival in relation to results of *BRAF* mutation analysis in 111 patients with peritoneal metastasis of colorectal or appendiceal origin

Patients whose tumor underwent molecular analysis had shorter survival than those not analyzed (Fig. 1a, *p* < 0.0001). In all, 101/111 received systemic chemotherapy whereas 10 were not treated. Oxaliplatin-based chemotherapy was used in 55 cases, 5-fluorouracil monotherapy in 9 subjects, whereas 37 patients received both oxaliplatin and irinotecan. In addition, 21 received the anti-EGFR antibody cetuximab and 21 received the VEGF inhibitor bevacizumab. Totally, 36 were treated with one line of chemotherapy, 32 with two lines, 23 with three lines, and 7 with four lines of systemic chemotherapy. Also, after exclusion of patients with appendiceal neoplasms and pseudomyxoma peritonei, there was still a difference between those analyzed and those not analyzed (Fig. 1b, *p* = 0.019). However, there was no difference in survival related to *KRAS* mutation (*p* = 0.126, Fig. 2a), whereas *BRAF* mutation was associated with shorter survival (Fig. 2b, *p* = 0.028). These results were also true when only those with CC0 were included in the analyses (data not shown). On multivariate analysis based on all 92 subjects with complete information about age, gender, PCI, CCS, *BRAF* mutation, and signet cell differentiation, CCS (*p* = 0.00001), signet ring cell differentiation (*p* = 0.00001), and *BRAF* mutation (*p* = 0.0021) emerged as the statistically significant variables associated with poor survival (Table 4). When the analysis was repeated and stratified based on the open and close procedure, the importance of CCS disappeared and only signet ring cell differentiation (*p* = 0.000002) and *BRAF* mutation (*p* = 0.0049) were predictive of survival.

**Table 4:** Risk for death in 92 patients with peritoneal metastasis from colorectal and appendiceal tumours scheduled for CRS and HIPEC, with BRAF mutation status available

	RR	95% CL of RR	*p*
Gender	1.021	0.707–1.475	0.9118
Age	0.998	0.982–1.014	0.7874
PCI	1.001	0.980–1.023	0.9063
CCS	1.583	1.319–1.899	0.000001
No signet cells	0.298	0.189–0.470	0.000001
*BRAF* mutation	4.412	1.714–11.315	0.0021

Associations between variables and survival analyzed with a multivariate Cox regression procedure. Risk estimates expressed as relative risk (RR) with 95% confidence limit (CL) and *p* value

## Discussion

The results of this study reveal that *BRAF* mutation is a negative prognostic marker in patients with peritoneal metastases from appendiceal or colorectal cancer scheduled for CRS and HIPEC. The proportion of *BRAF* mutated tumors in our study was 10 out of 92 (11%), which is in line with previous studies.^17^,^18^ In the present study, *BRAF* mutation was associated with shorter survival compared with *BRAF* wild type. This is in accordance with previous studies where *BRAF* mutation has been associated with poor clinical outcome in patients with colorectal hepatic metastases.^17^^–^20As shown in this study, which focuses on patients with colorectal peritoneal metastases, *BRAF* mutation indicated poor prognosis, with no patient surviving for more than 2 years. Moreover, when assessed using multivariate analysis, *BRAF* mutation emerged as a major determinant for short survival. This finding suggests that patients with *BRAF* mutation might benefit from a different therapeutic approach, such as upfront neoadjuvant treatment or palliative treatment using *BRAF* inhibitors. Protein kinase treatments are evolving rapidly, with vemurafenib as the first *BRAF* inhibitor on the market now indicated for metastatic malignant melanoma,^21^ and a combination treatment with MEK inhibitors has been shown to improve the response rate compared with *BRAF* monotherapy.^22^ However, metastatic colorectal cancer has not responded well to *BRAF* inhibitors used as monotherapy, and it is thought that *BRAF* inhibitors will have to be used together with other targeted drugs or chemotherapy. Several such clinical trials are ongoing.^23^

Mutation in *KRAS* was present in 51 out of 110 (46%) cases, which is somewhat higher than in patients with colorectal hepatic metastases.^24^^–^26 Moreover, in our study, mutated *KRAS* did not affect survival. This is contrary to patients with liver metastases.^24^^–^26 This difference underscores that patients with peritoneal metastases have a different clinical course and a unique biologic tumor behavior shown by the tendency to mucinous differentiation and superficial spread rather than hematogenous spread. The survival of patients with *KRAS* mutated tumors was actually longer than for those with *KRAS* wild-type tumors. Although the difference was not statistically significant, we can conclude that *KRAS* mutation is not a poor prognostic sign in peritoneal metastases.

The worse prognosis in subjects whose tumor was submitted for *KRAS* and *BRAF* mutation analysis compared with those not analyzed could be explained by a need for more antitumor treatment in addition to CRS and HIPEC, where patients with unfavorable prognostic signs are selected for mutation analysis. The most clinically important value of mutation analysis is to predict the effect of anti-EGF antibody treatment,^27^ which is common in neoadjuvant regimes prior to hepatic resection or in other situations when downsizing or downstaging is warranted. Since patients whose tumor was tested differed from those untested with respect to prognosis, subjects with mutations should be compared with those tested but found not mutated. This must be remembered when interpreting the results, but since a need for additional systemic chemotherapy is common, we believe the results are applicable to a large proportion of patients. Another limitation of this study is that the source of mutation analyses was either the primary tumor or peritoneal metastases. However, the concordance rate of *KRAS* and *BRAF* mutation analysis was 94% and 100% in a previous report,^10^ suggesting only a marginal influence of whether the sample for mutation analysis is taken from the primary tumor or the metastasis. A final limitation is that only *KRAS* and *BRAF* mutation was analyzed, since more extensive RAS mutation analysis was not in routine use during the study period.

The main prognostic factors in patients undergoing CRS + HIPEC because of CRC peritoneal surface malignancy are tumor burden according to PCI and radicality of cytoreduction result measured with the CC score. In addition, prior surgical score and presence of signet ring cell differentiation have been recognized as prognostic factors. Combinations of several variables as in the peritoneal disease severity score,^28^ the COREP score,^29^ and COMPASS score 30 have also proven to be of predictive value.

Few studies have addressed RAS status in peritoneal metastases. Massalou et al.^31^ observed that both *KRAS* and *BRAF* mutation were associated with mucinous differentiation but not clearly related to prognosis, while Jones et al.^32^ identified a subgroup of *BRAF* mutations outside codon 600 in patients with metastatic colorectal cancer linked with favorable prognosis. Finally, a recently published study found that both *KRAS* and *BRAF* mutation impaired overall survival after CRS and HIPEC.^11^

In conclusion, *BRAF* mutation is a marker of poor prognosis in patients with appendiceal and colorectal peritoneal metastases scheduled for treatment with CRS and HIPEC, whereas the survival outcome in subjects with *KRAS* mutated tumors does not differ from that in patients with *KRAS* wild-type tumors.
